# Preparation and Characterization of Ethylenediamine-Polyurea Microcapsule Epoxy Self-Healing Coating

**DOI:** 10.3390/ma13020326

**Published:** 2020-01-10

**Authors:** Yanxuan Ma, Yingrui Zhang, Jiatong Liu, Yi Sun, Yajie Ge, Xiaoning Yan, Jian Wu

**Affiliations:** 1 Department of Material Science and Engineering, School of Civil Engineering, Qingdao University of Technology, Qingdao 266033, China; zhangyingrui666@163.com (Y.Z.); liujiatong0303@gmail.com (J.L.); geyajie15030112@163.com (Y.G.); yxn1538563129@163.com (X.Y.); 2 School of Materials Science and Engineering, University of Science and Technology Beijing, Beijing 100083, China; sunyi271828@126.com; 3 Division of Advanced Nano-Materials and Division of Nanobionic Research, Suzhou Institute of Nano-Tech and Nano-Bionics, Chinese Academy of Sciences, Suzhou 215123, China; jwu2014@sinano.ac.cn

**Keywords:** ethylenediamine, polyurea, microcapsules, self-healing, anti-corrosion

## Abstract

Polyurea microcapsules with Ethylenediamine (EDA) as the core material were synthesized. A set of characterization methods, including optical and scanning electron microscopy (OM and SEM), the Fourier transform infrared (FTIR) spectroscopy and thermogravimetric analysis (TGA) were used to confirm the microcapsule morphology and chemical structures. The influence of emulsifier content and stirring rate on size and morphology of the microcapsules was investigated, and the self-healing performance of EDA-Polyurea microcapsule/epoxy coatings was evaluated by electrochemical impedance spectroscopy (EIS) measurements. The results showed that the microcapsules obtained had good spherical shape with a mean diameter of 0.54–0.70 μm. Compared with pure core material, the microcapsule showed excellent thermostability, and the content of core materials was up to 56.00 wt%. The epoxy coating with 5.0 wt% EDA-Polyurea microcapsules achieved average corrosion resistance efficiencies of 90.00%, significantly enhancing the capability of the scratched coating to resist external corrosion.

## 1. Introduction

As it is convenient for construction and excellent for anti-corrosion, coatings are widely used in metal protection. However, when affected by the external forces, the internal structure and properties of coating materials can be easily degraded with micro-cracks of different sizes. Furthermore, the continuous generation and development of micro-cracks inside will eventually cause cracking or breakage of the coating materials at the macroscopic level, which in turn will lead to severe damage to the mechanical properties of the coating. In addition, these internally generated micro-cracks are difficult to restore from the outside using existing methods. To solve this problem, self-healing coatings have been studied as protective materials and exploited extensively in the past few years [1,2,3,4]. Self-healing coatings can respond quickly to environmental changes and repair material damage automatically. Inspired by White [5], who was famous for inventing the self-healing microcapsule system, researchers have aimed to create self-healing systems that can make the fracture repair more sustainable. When the self-healing system is stimulated by the formation of a crack, the encapsulated healing materials will be ruptured and then released.

Encapsulation of healing agents has been demonstrated as a very effective method for fixing cracks completely with hardener, thus avoiding the corrosion associated with aggressive substances [6,7,8]. To date, the literature has revealed many compounds, such as epoxy resin [9,10,11], linseed oil [12,13,14,15], formaldehyde and urea formaldehyde resin [12,16,17], that have been used for the self-healing microcapsules. Despite some advantages [18,19,20], such as cheap and strong bonding, faster curing rate and more complex self-repair system, a complete theoretical basis and superb synthesis process have not yet been developed for epoxy resin systems, which leads to certain limitations. Liquid isocyanates, one of the most promising self-healing materials, nowadays, easily reacts with moisture and has been becoming more and more popular in the research of microcapsules [21,22]. Wu G. et al. [23]. successfully synthesized silica/polyurea hybrid microcapsules encapsulated with hexamethylene diisocyanate (HDI) by using interfacial polymerization and sol-gel methods. The results showed that the diameter and shell thickness were linearly related to the agitation rate and the microcapsules exhibited excellent heat resistance. Haghayegh et al. [24] prepared and characterized a new isocyanate-terminated prepolymer based on IPDI as a healing agent (BIH), and the salt spray test showed that the BIH-containing polyurethane microcapsules had superior performance in corrosion resistance compared with single IPDI monomer microcapsules. Huang et al. [25] synthesized polyurethane (PU) microcapsules coated with hexamethylene diisocyanate (HDI). The reaction time and temperature, as well as the effect of surfactant content and stirring rate on the microcapsules, were studied. The anti-corrosion properties of epoxy coatings containing microcapsules were tested. The preliminary results showed that single-component self-healing coatings had great potential in corrosion control. With respect to one-part catalyst-free self-healing systems, these studies have made great progress.

Since little attention has been directed towards the preparation of microcapsules containing amine curing agent for diisocyanate self-healing systems, in this study, the work is devoted to the exploration of polyurea microcapsules with ethylenediamine, which are able to survive the mixing process without any particular protection and break upon the appearance of cracks to react with diisocyanate. Additionally, this study illustrates details regarding the preparation procedures of the obtained microcapsules, particularly the influence of the droplet size of the prepolymer and ethylenediamine emulsion on the microcapsule morphology. Also, unlike most previous studies, this paper aims to show clear visual observations of the self-healing process and the performance of the modified coating using effective evidence. Furthermore, there is no need to use an easily deactivated and expensive catalyst in the microcapsule synthesis method, which effectively solves problems such as the low rate of solidification reaction, film formation speed, and failure in crack healing. Finally, the optimal experimental synthesis parameters for novel EDA-Polyurea microcapsules were able to be achieved, and the composite polymer coatings were endowed with effective self-healing potentiality and long-term anticorrosion properties.

## 2. Materials and Methods

### 2.1. Materials

Isophorone diisocyanate (IPDI) and Polyether amines ethylene (Poly bis) with a purity of 99.00 wt%, used as shell material for polyurea microcapsules, were purchased from Shanghai Aladdin Biochemical Technology Co., Ltd. (Shanghai, China). Ethylene-diamine (EDA, 99.00 wt%), as a core material, was supplied by Tianjin Damao Chemical Reagent Factory (Tianjin, China). Sodium dodecyl benzene sulfonate (SDBS), as the emulsifier, was obtained from Shanghai Aladdin Biochemical Technology Co., Ltd. (Shanghai, China). Cyclohexane, as the oil phase, was supplied by Shanghai Aibi Chemical Reagent Co., Ltd. (Shanghai, China). Bisphenol A epoxy resins Epon 828 (equivalent of epoxy: 185–192) and Epikure 3164 (equivalent of curing agent: 256) were purchased from Danbao Resin Co., Ltd. (Chuzhou, China). All chemicals in this study were used without further purification unless otherwise specified.

### 2.2. Synthesis of EDA-Polyurea Microcapsules

**Figure 1:**
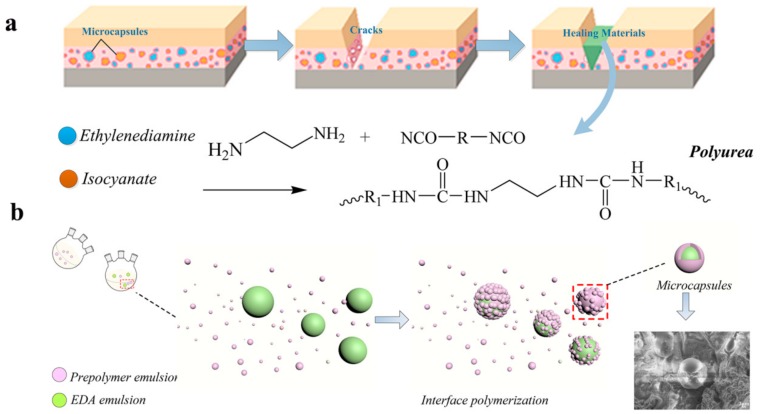
(**a**) Molecular structure and self-healing mechanism of EDA-Polyurea microcapsule; (**b**) Preparation process of EDA-Polyurea microcapsule.

Polyurea microcapsules encapsulated with EDA were synthesized by carrying out an interfacial polycondensation reaction at the interfaces. Figure 1a shows the molecular structure and self-healing mechanism of EDA-Polyurea microcapsule, and Figure 1b is the preparing process of the EDA-Polyurea microcapsule. It follows a typical experimental procedure: firstly, the EDA emulsion was prepared by adding 110 g cyclohexane and a certain amount of SDBS to a round-bottom flask, which was then agitated for 60 min using a magnetic stirrer at 2000 rpm and 25 °C. Then, 9.6 g EDA was dropped into the oil solution to form a stable suspension with the speed around 1000–2000 rpm. Meanwhile, the prepolymer emulsion containing 3.0 g IPDI and 9.0 g Polyether amines was dissolved in 66 g cyclohexane and SDBS mixture, and then agitated for 40 min at 1500 rpm and 25 °C. Then, at 1000–2000 rpm, the emulsion of prepolymer was added into the emulsion of EDA to stimulate the interfacial polycondensation reaction, forming a polyurea shell at the interface of the core suspension at 60 °C for 3 h with constant stir. The resultant microcapsules were washed with deionized water, filtered and air-dried at 25 °C for 24 h before further analysis.

### 2.3. Morphology of the Microcapsules

The microcapsule synthesized with different content of emulsifiers and various stirring speed were observed under an optical microscope (OM, UMT203, Aopu Optoelectronic Technology Co., Ltd., Chongqing, China). The surface morphology and topography of the microcapsules were studied by using scanning electron microscopy (SEM, ULTRA-55, ZEISS Co., Ltd., Jena, Germany). For the SEM measurement, the samples were painted with a gold layer to avoid charging in the process of SEM measurement [26,27,28].

### 2.4. FTIR Spectroscopy of the Microcapsules

During the process of microcapsules synthesis, the FTIR spectra of EDA-Polyurea microcapsules and control groups including the shell and pure EDA were measured with respect to the urea-bond functional groups by using Fourier transform infrared spectroscopy (Nicolet8700, Thermo Fisher Scientific, Waltham, MA, USA) in a wave number range of 500–4000 cm^−1^ [29,30].

### 2.5. Thermal Analysis and Core Content of the Microcapsules

TGA (TA Instruments Inc; STA409PC, Netzsch Scientific Instruments Trading Co., Ltd., Selb, Germany) was used to analyze the thermal stability and core content of the prepared microcapsules by comparing the TGA and DTG traces of microcapsules, core and shell materials. The microcapsules were heated from 25 to 650 °C at a rate of 20 °C/min in a nitrogen atmosphere with a gas purge of 40 mL/min. The core content of the microcapsules was roughly calculated by the decreased peak of the weight loss curve [12,29,31].

### 2.6. Preparation and Characterization of the Self-Healing Coating

EIS measurements (p4000A, Ametek Group Advanced Measurment Technology, Princeton, NJ, USA) have conventionally been used to evaluate the performance of self-healing coatings [22,29]. Before the EIS measurement, the Q235 steel samples (50 mm × 50 mm) were coated by a series of modified epoxy resins with different contents of synthesized microcapsules (0.0 wt%, 2.0 wt% and 5.0 wt%). Then, these samples were cured at 25 °C for 72 h. Impedance measurements were carried out in a frequency range of 100 kHz to 0.1 Hz with different immersing times in seawater. To allow enough repair agents to heal the damage, as many microcapsules as possible should be incorporated without affecting the performance of the coating. Meanwhile, SEM was employed to observe the scratched surface morphology of the self-healing coating to provide intuitive information of the anti-corrosion process of the coatings [32,33,34].

## 3. Results and Discussion

### 3.1. Influence of Emulsifier Content on the Microcapsules

The study of emulsification of the core material plays a crucial role in the success of the experiment. In the emulsification process, external forces work on the system, forcing the core material to disperse in the solvent, so the amount of emulsifier has great influence on the size and distribution of the final prepared microcapsules. In this study, SDBS was used as an emulsifier, and the influence of the amount of emulsifier on the morphology of microcapsules was investigated by changing the amount of emulsifier. The amount of SDBS used was 5.0 wt%, 10.0 wt%, 15.0 wt% and 20.0 wt% of the core material, respectively.

**Figure 2:**
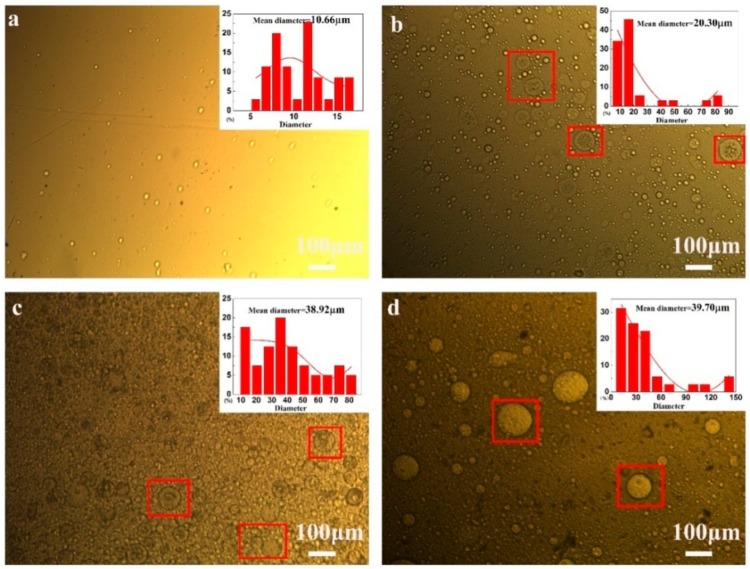
Emulsion with different contents of emulsifying agent: (**a**) 5.0 wt% SDBS; (**b**) 10.0 wt% SDBS; (**c**) 15.0 wt. % SDBS; (**d**) 20.0 wt% SDBS.

Figure 2 shows the emulsion of polyurea microcapsules with different contents of emulsifying agent. In Figure 2a, when the amount of emulsifier was 5.0 wt%, the prepared microcapsules had a mean diameter of 10.66 μm, which was mainly because the SDBS in lower content was insufficient to protect the small microcapsules’ aggregation, resulting in low production of microcapsules [35]. When the amount of emulsifier was 10.0 wt% (Figure 2b), the number of microcapsules gradually increased, and the distribution was more uniform, with an average size of 20.30 μm. This phenomenon can be explained by the fact that, with the increase of emulsifier content, the core material droplets were tightly coated with emulsifier molecules, reducing the probability of collision between droplets. When the emulsifier content was continuously increased to 15.0 wt%, the particle size distribution became relatively more concentrated, and the average size of the microcapsules increased to around 38.92 μm. However, when the amount of emulsifier exceeds 20.0 wt%, the size of microcapsules ranges from 10 to 90 μm with a mean diameter of 39.70 μm, with an uneven distribution of microcapsules. The main reason for this is that the surface tension at the interface of the two phases inconspicuously decreases with the increase of the amount of emulsifier, resulting in the demulsification of SDBS detaching from the surface of the rejuvenator, causing a decrease in the number of microcapsules and an increase in particle size [27]. Taking particle size distribution and average particle size into consideration, the optimal amount of emulsifier SDBS is 15.0 wt%.

### 3.2. Influence of Rotating Speed on the Microcapsules

The stirring rate has a great effect on the synthesis and size of the polyurea microcapsules. To obtain perfect microcapsules, what needs to be ensured is that the size of the prepolymer droplets is smaller than that of the core EDA emulsion droplets. In this study, three groups of comparative experiments were set up with the prepolymer droplets and EDA droplets as the research objects, changing the rotation speed during emulsification to achieve the required experimental parameters.

**Figure 3:**
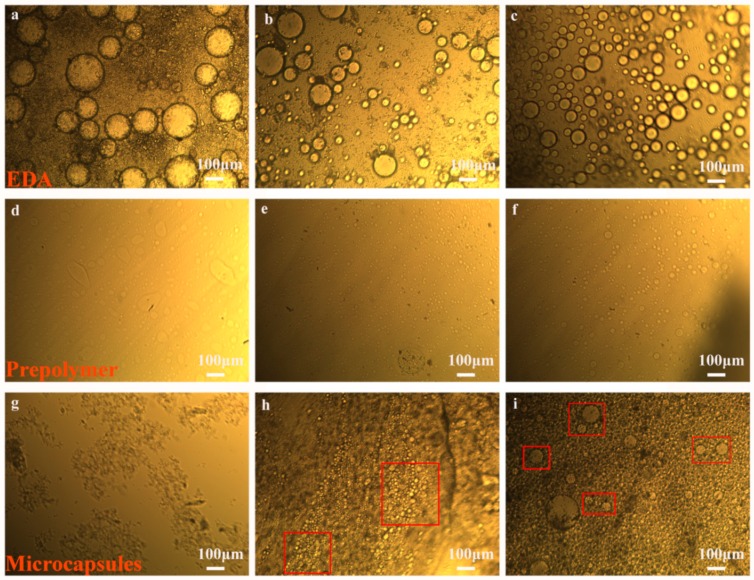
OM morphology of the microcapsules with different stirring rates: microcapsules (**g**) were obtained with EDA (**a**) at 1000 rpm and polymer (**d**) at 1500 rpm; microcapsules (**h**) were obtained with EDA (**b**) at 1500 rpm and polymer (**e**) at 2000 rpm; microcapsules (**i**) were obtained with EDA (**c**) at 2000 rpm and polymer (**f**) at 2000 rpm.

In general, the EDA and prepolymer droplet size gradually became smaller as the rotation speed increased. Navarchian et al. [32] prepared poly (methyl methacrylate) (PMMA) microcapsules containing linseed oil microcapsules at three different stir rates: 1000, 600 and 300 rpm. They found that when the agitation rate was changed from 300 to 1000 rpm, the size of microcapsules became narrower. The result of emulsion and microcapsule droplets is consistent with previous research conducted by Behzadnasab et al. [1] and Lang et al. [12]. As shown in Figure 3a,d,g, spherical microcapsules cannot be obtained with the emulsification speed of the EDA at 1000 rpm and the prepolymer at 1500 rpm. The reason for this phenomenon may be that during the emulsification process, the droplets of the prepolymer emulsion are not uniform, and the polymerization reaction cannot occur at the interface with the EDA droplets. In Figure 3b,e,h, when the rotational speeds of the EDA and prepolymer increase to 1500 rpm and 2000 rpm, respectively, spherical microcapsules with a particle size of approximately 10 μm can be obtained. If the rotational speed of the EDA emulsion is continuously increased to 2000 rpm, concentrated microcapsules can be obtained with an average size of around 15 μm (Figure 3c,f,i). Considering the effect of stirring speed on microcapsules, microcapsules (i) synthesized with EDA emulsion 2000 rpm (c) and polymer emulsion 2000 rpm (f) showed good morphology and size distribution.

**Figure 4:**
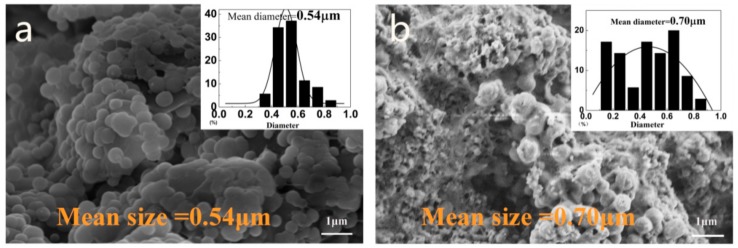
SEM Morphology of EDA-Polyurea microcapsules. (**a**) microcapsules with a mean size of 0.54 μm (**b**) microcapsules have regular spherical shapes with a mean size of 0.70 μm.

Under optimum experimental conditions, the surface morphology (Figure 4) of the polyurea microcapsules was studied by SEM. Figure 4a shows that the synthesized microcapsules have regular spherical shapes with a mean size of 0.54 μm, and Figure 4b shows larger-sized microcapsules with a mean size of 0.70 μm, which is different from those observed under the optical microscope (around 38 μm). This can be explained by the fact that the microcapsules under the optical microscope are in an emulsion state, and as the microcapsules are centrifuged, filtered, and cured, some of the larger microcapsules are broken due to instability. In addition, the phenomenon of adhesion in microcapsules (Figure 4b) may be caused by the outflow of EDA due to rupture of the microcapsules. Therefore, methods of improving the stability of microcapsules in the curing process need to be further explored.

### 3.3. Chemical Structure of the Microcapsules

**Figure 5:**
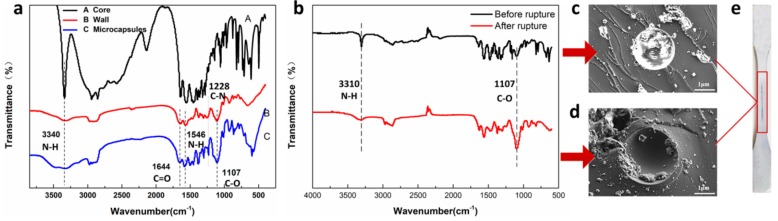
(**a**) FTIR spectrum of core, shell and microcapsules; (**b**) FTIR spectrum of microcapsules before and after rupture; (**c**) microcapsule before rupture; (**d**) microcapsules after rupture; (**e**) coating example with crack.3.4. Thermostability and Core Content of the Microcapsules.

The chemical structures of the core material, shell material, and the obtained microcapsules were characterized by Fourier Transform Infrared Spectroscopy (FTIR). As shown in Figure 5a, it was found that the typical signal at 3340 cm^−1^ is attributed to N–H stretching vibrations, and signal peaks at 1644 cm^−1^, 1546 cm^−1^ and 1288 cm^−1^ are assigned to C=O stretching vibrations and N–H, C–N stretching band. The above bands confirm the presence of the –NHCONH– structure in the microcapsules, which also clearly confirms the shell structure consisting of polyurea. Furthermore, according to the FTIR spectra of Lee et al. [30], there is no obvious peak in the FTIR spectrum at 2270 cm^−1^ related to the free –NCO groups, which indicates complete reaction of IPDI. Meanwhile, it also proves that the excess core material EDA is successfully encapsulated. It must be noted that the N–H stretching peak at 3340 cm^−1^ becomes very weak due to hydrogen bonding, and C–O were observed at 1107 cm^−1^, which demonstrates that the Polyurethane is produced due to side reactions between isocyanate and water vapor. Therefore, the synthetic method is effective for encapsulating EDA of core materials during polymerization procedure.

In addition, the self-healing mechanism of the composite coatings can be characterized by FTIR and SEM. Figure 5c,d presents the morphology of the microcapsules before and after rupturing in the epoxy coating (Figure 5e), respectively (microcapsules shown in Figure 5c,d were produced in the same batch, but they are not the same one), and it can be found that some reaction products remain in the microcapsule cavity after rupture. Combined with Figure 5b, it can be seen that after the microcapsules break, the N–H stretching vibrations in 3310 cm^−1^ are weakened, and the signal peak in 1107 cm^−1^ related to C–O is strengthened, indicating the consumption of the core material ethylenediamine when the capsule breaks, as well as the formation of polyurethane as a by-product.

**Figure 6:**
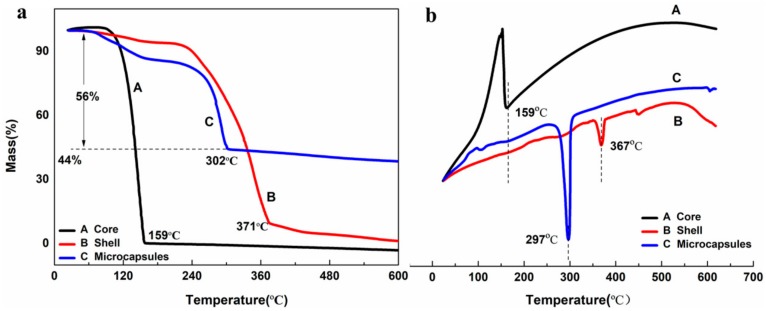
(**a**) TG and (**b**) DSC curves of the core, shell and microcapsules.

During the service life, microcapsules must have good heat resistance to maintain the effectiveness and stability of the core materials [36,37]. To identify thermal stability and the content of core material (EDA) in the microcapsules, thermogravimetric analysis was used to evaluate the properties of the core material, shell material and microcapsules. The weight loss curves of the synthesized microcapsules along with pure shell material and pure EDA are shown in Figure 6a. It can be observed that the pure core material decomposes from 83 °C and completes this decomposition at 159 °C, and the rate at which weight was lost was extremely fast. From the curve of the shell material, it can be found that with high heat resistance, the initial decomposition temperature of the shell material is 227 °C. Meanwhile, the curve of the microcapsules showed that the mass slowly decreased with increasing temperature until the complete decomposition of the core material was achieved at 302 °C, which also indicated that the core fraction encapsulated by microcapsules was approximately 56.0 wt%. The previous research reported by Lang et al. [12] showed that the encapsulated core percentage was in the range of 76 to 82 wt% for the microcapsules obtained under different stirring rates, and Ye et al. [31] found that encapsulation ratio of poly (urea-formaldehyde) microcapsules was 90.42%. In addition, isophorone diisocyanate (IPDI) microcapsules prepared by Attaei et al. [29] showed that the core content of epoxy ester was 76 wt%. Compared with these results, the main reason for the relatively low content of the core material in our experiment was that EDA was a volatile liquid, and a small amount of EDA was volatilized during the synthesis.

The extremely high content of EDA in the microcapsules ensures that enough curing agent is able to be released and filled in cracks. As shown in Figure 6b, the DSC experiment of the core material showed one sharp endothermic peak at around 159 °C, and the derivative peak of shell material was found at 367 °C, both of which are highly consistent with complete decomposition temperature in TGA curves. What is important is that one sharp peak of microcapsules shows not only successful encapsulation of EDA by shell materials, but also the significant increase in thermal properties. Through the TGA and DSC curves, it can be noted that the decomposition rate of the core material is significantly reduced under the coating of the shell material. Therefore, the microcapsules synthesized by this method possess an extremely high content of core material and excellent thermal performance, which has an important effect on self-healing performance.

### 3.4. Electrochemical Corrosion Resistance of the Self-Healing Coating

**Figure 7:**
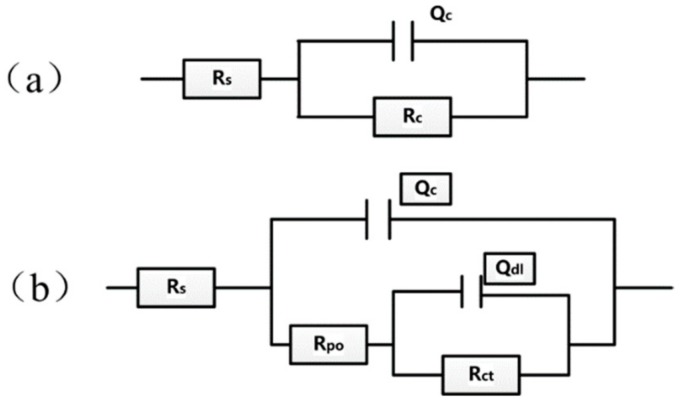
Equivalent circuit models used to fit the experiment impedance data of scratched coating immersion in seawater. (**a**) Equivalent circuit model in the early stage of immersion (**b**) Equivalent circuit model in the middle and late stage of immersion.

EIS provides a relatively reliable method for studying the occurrence and development of electrochemical reactions at organic coating/metal interfaces [10,38,39,40]. The Nyquist and Bode plots, with different concentrations of microcapsules and immersing time, were obtained after scratching. At the early stage of solution penetration into the coating, the equivalent circuit (Figure 7a) can be used to simulate the EIS parameter. R_s_, R_c_ and Q_c_ represent the solution resistance, coating resistance and capacitance of the scratched coating, respectively. When the penetration of the electrolyte solution into the coating reaches saturation within a certain period of time, a corrosion microbattery will be formed at the coating/base metal interface. In that case, the equivalent circuit in Figure 7b can be employed, and the EIS has two time constants: One was attributed to the coating capacitance Q_c_ and the microporous resistance R_po_ of the coating surface, and the other was due to the electric double layer capacitance Q_dl_ and charge transfer resistance R_ct_. The EIS data shown in the former 72 h were fitted with the equivalent circuit (Figure 7a), whereas the EIS data shown after the 72 h, fitted with the equivalent circuit, are shown in Figure 7b. The definition of the impedance of the constant phase element (CPE) is as follows:

(1) $$Z_{\mathrm{CPE}}=\frac{1}{Y_{0}{\left(\mathrm{jw}\right)}^{n}}$$

where Y_0_ represents the CPE constant (F·cm^−2^ s^n−1^ or s^n^ Ω^−1^·cm^−2^) and n is the index of it, j is a constant equal to √−1, and ω refers to the angular frequency. CPE has different meanings with the change of n. When the n = 0, Z_CPE_ = R, the CPE represents resistance, when n = 1, Z_CPE_ = C, it represents capacitance, while when n = −1, Z_CPE_ = L, it represents inductance. In particular, when n = 0.5, it represents Warburg impedance. The equation for converting CPE constant Y_0_ to double capacitor C_d1_ is as follows:

(2) $$C_{\mathrm{dl}}=Y_{0}{\left(w_{\max}\right)}^{n-1}$$

where ω_max_ is the maximum angular frequency of the imaginary part of the impedance. The fitting results of the experimental EIS are listed in Table 1 and Table 2. As the content of microcapsules in the coatings increases, the corrosion resistance gradually increases. The theoretical estimates of corrosion resistance efficiencies (CRE) of the coating can be calculated through the following equation:

(3) $${\mathrm{CRE}}_{\mathrm{EIS}}\left(\%\right)=\frac{R_{\mathrm{ct}}^{\prime }-R_{\mathrm{ct}}}{R_{\mathrm{ct}}^{\prime }}=1-\frac{R_{\mathrm{ct}}}{R_{\mathrm{ct}}^{\prime }}$$

where R_ct_ and R′_ct_ represent the charge transfer resistance of the coating matrix without and with microcapsules, respectively. In addition, in order to simplify these samples, the samples with microcapsules in different contents will be defined as MX. M stands for the microcapsules, and X stands for the microcapsule concentration. For example, M5 means that the coating sample incorporated 5.0 wt% microcapsules. For coatings containing different contents of microcapsules, the measurements were repeated with 4 samples to ensure reproducibility.

**Figure 8:**
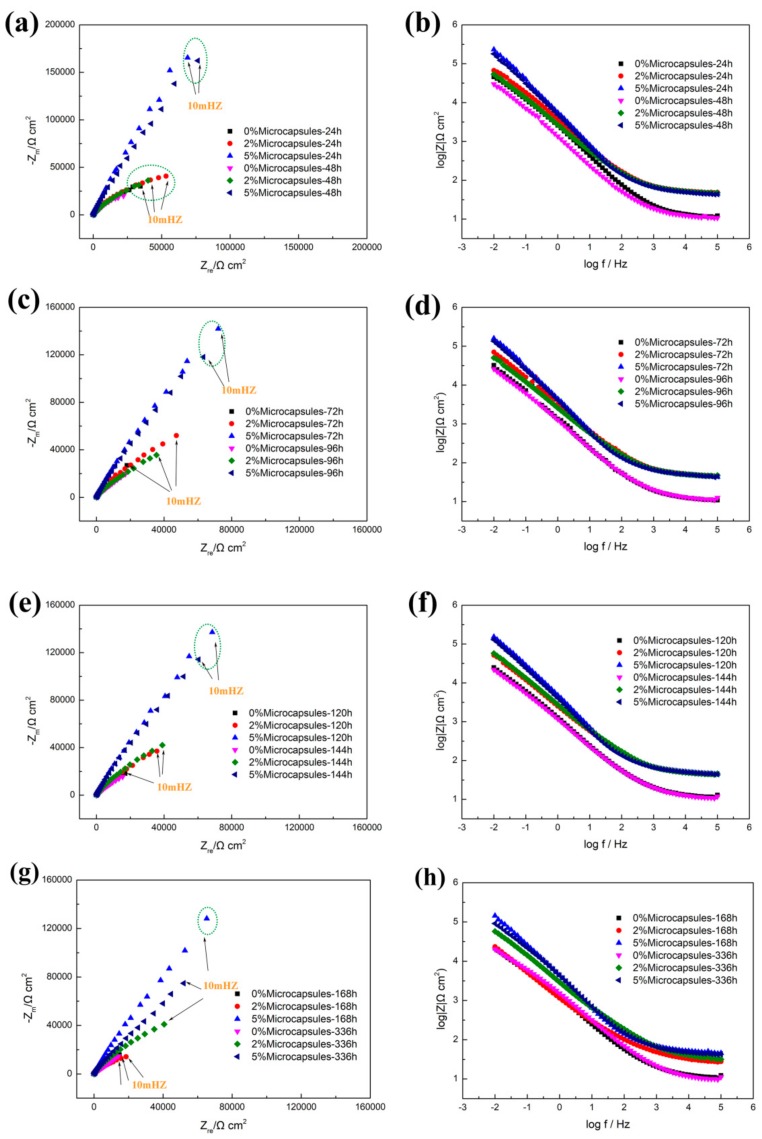
Nyquist and Bode plots of coatings with different contents of microcapsules (0.0 wt%, 2.0 wt% and 5.0 wt%) after being scratched and immersed in seawater for different times. (**a**) Nyquist plots after 24 and 48 h (**b**) Bode plots after 24 and 48 h (**c**) Nyquist plots after 72 and 96 h (**d**) Bode plots after 72 and 96 h (**e**) Nyquist plots after 120 and 140 h (**f**) Bode plots after 120 and 140 h (**g**) Nyquist plots after 166 and 336 h (**h**) Bode plots after 166 and 336 h.

**Figure 9:**
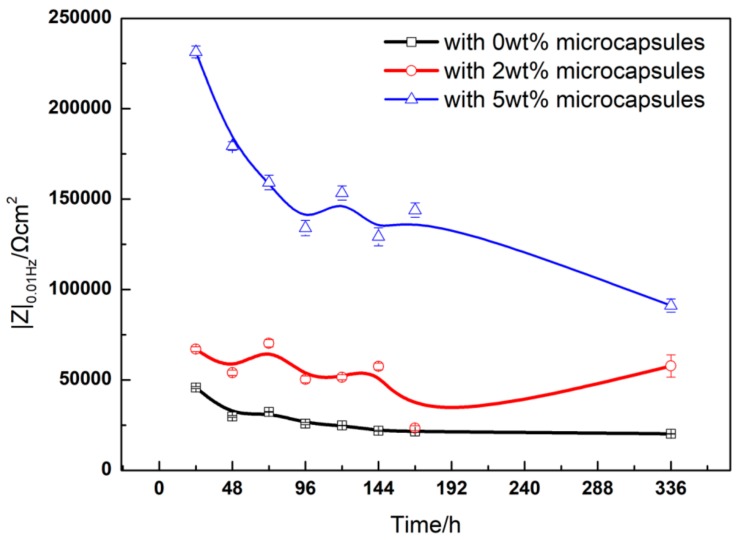
Impedance modulus (f = 0.01 Hz) of the scratched coating after immersion in seawater.

Generally, Impedance modulus (|Z|) at a frequency of 0.01/0.005 Hz is applied to evaluate the coating resistance. The |Z|_0.01Hz_ value of the alkyd varnish coatings AVC containing isophorone diisocyanate (IPDI) increased from 0.782 to 1.515 MΩ·cm^2^ [22], indicating the barrier properties of modified coatings, and this figure for self-healing epoxy coatings presented by Attaei et al. [29] showed a similar trend, rising from 1 GOhm·cm^2^ to 10 GOhm·cm^2^ after 35 days’ immersion. Similarly, in this study, Figure 8b,d shows the Bode plots of scratched coatings with different microcapsule contents after immersion for 24 to 96 h. It can be seen that the impedance magnitude of M5 is much higher compared to that of M2 and M0 throughout the whole process. It should be noted that in Figure 8f,h, the impedance (|Z|) of M2 increases more obviously than it did in the previous 24 h, indicating the slightly on-going self-healing process. Combined with Figure 9, when M5 is immersed in seawater for 24 h, |Z|_0.01Hz_ is 2.31 × 105 Ω·cm^2^, and after 72 h, 120 h and 144 h, it decreased by 31.20%, 42.08% and 33.71%, respectively. At 336 h, |Z|_0.01Hz_ is reduced to 9.10 × 10^4^ Ω·cm^2^. It can be clearly seen that the drop in the ratio between 72 and 144 h was very small, and the increase in the ratio after 120 h proved the occurrence of the self-repair process. With an initial |Z|_0.01Hz_ of 6.70 × 10^4^ Ω·cm^2^ at 24 h, M2 showed a similar trend. Moreover, the |Z|_0.01Hz_ value of M2 reached the maximum peak value of 7.03 × 10^4^ Ω·cm^2^, which shows an increase of 4.93% with respect to the initial value after 72 h immersion, and reaches another peak value of 5.74 × 104 Ω·cm^2^, which shows an increase of 11.47% with respect to 120 h after 144 h immersion. However, the |Z|_0.01Hz_ value of M0 maintains a decreasing trend from the initial 4.57 × 10^4^ to the final 2.02 × 10^4^ Ω·cm^2^, with a rate of decrease of 29.29% and 52.14% after immersion for 72 and 144 h, respectively.

The values of the impedance parameters of coated samples with 0.0 wt%, 2.0 wt%, 5.0 wt% microcapsules after immersion in seawater for 24 to 336 h were recorded. As shown in Table 1, the R_c_ values of the scratched coating with 5.0 wt% microcapsules was significantly higher than those with 2.0 wt% and 0.0 wt% microcapsules. In the initial 72 h, the only decreases from 1.95 × 10^6^ to 9.74 × 10^5^ Ω·cm^2^ indicate that the self-healing microcapsules effectively prevented the penetration of electrolyte solution into the substrate during the initial corrosion time. It should be noted that the R_c_ value of the M2 increased from 1.27 × 10^5^ to 2.13 × 10^5^ Ω·cm^2^, proving that the self-healing process occurred during the period of corrosion. Moreover, as shown in Table 2, there are obviously increasing trends of the R_c_ value in the samples of M5 and M2. The R_c_ value of M5 increases from 8.04 × 10^5^ to 9.20 × 10^5^ Ω·cm^2^, reaching a peak of 1.15 × 10^6^ Ω·cm^2^ at 120 h. Meanwhile, the R_c_ value of M2 shows a similar trend, increasing from 1.45 × 10^5^ to 2.13 × 10^5^ Ω·cm^2^ and reaching a peak of 1.91 × 10^5^ Ω·cm^2^ at 144 h. The increasing trend of R_c_ value in M2 and M5 demonstrates that the self-healing microcapsules can improve the corrosion resistance of the scratched coating. Moreover, the results of the corrosion resistance efficiencies are shown in Table 2 after immersion for 96 h. It can be found that the CRE increases with increased microcapsule content, and that the charge transfer resistance of the coating containing 5.0 wt% microcapsules was much higher than that of the blank coating. The highest CRE values of M5 were over 90.00%, reaching a value peak of 94.36% after immersion for 120 h, demonstrating remarkable corrosion resistance ability and excellent self-healing performance. The CRE values of M2 range from 49.52% to 77.13%. The performance of self-healing of M2 was not as significant as that of M5, which was attributed to the low content of microcapsules, which was not able to thoroughly heal the scratch and prevent the corrosion. Compared with M0, which lost the ability to prevent corrosion completely as R_c_ reached values lower than 4.23 × 10^4^ Ω·cm^2^, it can be concluded that the sample with 5.0 wt% microcapsules shows excellent corrosion resistance of 3.31 × 10^5^ Ω·cm^2^ until 336 h, proving that the microcapsules can release the healing agent and repair the scratch. Therefore, it is a protective method for enhancing the coating with self-healing microcapsules [33,41,42].

**Figure 10:**
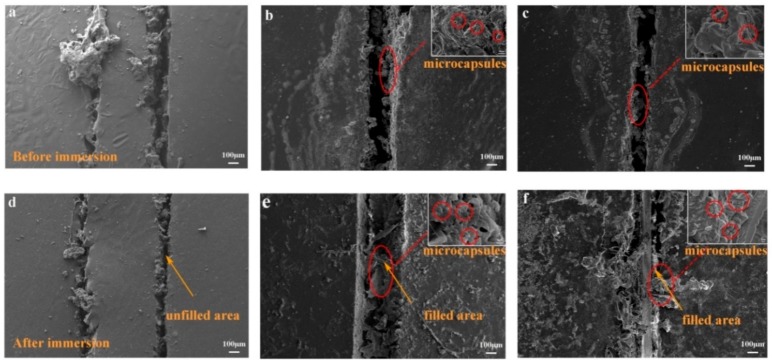
SEM morphology of the scratched coatings with different contents of microcapsules before and after immersion in seawater for 366 h ((**a**,**d**) pure epoxy coatings; (**b**,**c**) embedded with 2.0 wt% microcapsules; (**c**,**f**) embedded with 5.0 wt% microcapsules).

Artificial scratches are widely used in observing the self-healing ability and anticorrosion properties of composite coatings due to its convenience. The self-healing performance of the scratched coating embedded with different microcapsules content was evaluated by SEM [43]. Zhang et al. [33] showed that the micro-cracks in self-healing coatings were automatically covered and healed by healing agents compared to the control sample. As Li et al. [34] reported, a narrow gap was left after 15 days, but irregular edges were presented around the scratched area. Compared with them, the self-repairing microcapsules in this study can achieve excellent self-healing performance after 366 h. To detect the self-healing properties of microcapsules in corrosive environments, the prepared coatings were immersed in a NaCl solution for 366 h. In Figure 10, it can be obviously seen that the scratched areas of the coatings (Figure 10b,c) were partly distributed with microcapsules. After immersion for 366 h, the crack on the pure epoxy coating (Figure 10d) still remained unfilled and unable to resist the invasion of the corrosive medium. However, the crack on the coating containing the self-healing microcapsules was partially filled by the released healing agent from the ruptured microcapsules [44]. According to the micrographs of the scratched area of the coatings embedded with different microcapsules (Figure 10e,f), the coating filled with self-healing microcapsules greatly repaired the crack and exhibited excellent anti-corrosion performance prior to immersion. The reason for this can be attributed to the fact that the microcapsules were ruptured by the cracks and immersion, releasing large amounts of reactive isocyanates and EDA at the scratched area. Then, the released materials reacted to form a cross-linked polyurea, greatly preventing the spread of harmful medium. All in all, the self-healing process of microcapsules was confirmed by the results of SEM micrographs and EIS parameters.

## 4. Conclusions

In summary, spherical curing agent microcapsules with EDA as core material were obtained by interfacial polymerization in an oil-in-oil emulsion, and the self-healing property and anticorrosion performance of EDA-Polyurea-based coatings were significantly enhanced, enabling coatings to further meet the protection needs of marine engineering and showing great potential for practical marine applications. Three bullet points can be drawn as follows:

1. The effect of emulsifier content on the morphology distribution of microcapsules showed that the optimal amount of emulsifier SDBS was 15.0 wt%. Meanwhile, in order to obtain well-formed and evenly distributed microcapsules, the rotational speed of the EDA emulsion and prepolymer emulsion should both be set at 2000 rpm.
2. The FTIR spectrum indicated that the microcapsules were composed of both EDA (core materials) and Polyurea (shell materials). The mean diameter and core fraction of the final microcapsules were 0.54–0.70 μm and 56.00%, respectively. Moreover, compared with pure core material, the EDA encapsulated by microcapsules had a remarkably improvement in thermal stability, with the initial complete decomposition temperature of 159 °C increasing to the final 302 °C.
3. For the accelerated corrosion process, the scratched epoxy coatings coated with 5.0 wt% microcapsules exhibited excellent corrosion resistance, reaching 93.57% of self-healing efficiency after immersion for 366 h in seawater at 25 °C. The self-healing process of cracks was observed by SEM, the cracks in M5 were filled with a huge number of microcapsules, providing a great barrier for the coating, with results that were highly consistent with EIS data, indicating great potential for the use of the obtained microcapsules in self-healing coatings for corrosion resistance.

## Figures and Tables

**Table 1:** EIS parameters of the scratched coating immersing in seawater.

Time(h)	Content (wt%)	R_s_ (Ω·cm^2^)	Q_c_(F·cm^−2^)	R_c_(Ω·cm^2^)
24 h	0.0	12.02	1.06 × 10^−4^	8.16 × 10^4^
	2.0	51.38	7.24 × 10^−5^	1.27 × 10^5^
	5.0	51.56	4.03 × 10^−5^	1.95 × 10^6^
48 h	0.0	11.82	1.91 × 10^−4^	6.83 × 10^4^
	2.0	49.57	1.03 × 10^−4^	1.50 × 10^5^
	5.0	51.15	4.75 × 10^−5^	1.19 × 10^6^
72 h	0.0	12.27	1.63 × 10^−4^	6.71 × 10^4^
	2.0	48.83	7.83 × 10^−5^	2.13 × 10^5^
	5.0	51.38	5.28 × 10^−5^	9.74 × 10^5^

**Table 2:** EIS parameters of the scratched coating with immersion in seawater.

Time (h)	Content (wt%)	R_s_ (Ω·cm^2^)	Q_c_ (F·cm^−2^)	R_po_ (Ω·cm^2^)	Q_dl_ (F·cm^−2^)	R_ct_ (Ω·cm^2^)	CRE (%)
96 h	0.0	11.17	1.92 × 10^−4^	72.39	2.61 × 10^−5^	7.32 × 10^4^	–
	2.0	46.31	9.52 × 10^−5^	1312	1.39 × 10^−5^	1.45 × 10^5^	49.52
	5.0	45.05	4.25 × 10^−5^	99.71	1.65 × 10^−5^	8.04 × 10^5^	90.90
120 h	0.0	11.35	1.97 × 10^−4^	87.62	2.34 × 10^−5^	6.49 × 10^4^	–
	2.0	45.55	9.38 × 10^−5^	1379	1.76 × 10^−5^	1.66 × 10^5^	60.90
	5.0	46.15	4.12 × 10^−5^	127.20	9.63 × 10^−6^	1.15 × 10^6^	94.36
144 h	0.0	10.69	2.10 × 10^−4^	68.26	3.19 × 10^−5^	5.72 × 10^4^	–
	2.0	44.77	8.66 × 10^−5^	1478	1.46 × 10^−5^	1.91 × 10^5^	70.05
	5.0	45.39	4.36 × 10^−5^	99.38	1.62 × 10^−5^	7.94 × 10^5^	92.80
168 h	0.0	10.66	2.24 × 10^−4^	108.6	1.13 × 10^−5^	5.92 × 10^4^	–
	2.0	37.35	8.09 × 10^−5^	1018.8	1.34 × 10^−5^	1.89 × 10^5^	68.68
	5.0	46.62	4.36 × 10^−5^	118.4	1.23 × 10^−5^	9.20 × 10^5^	93.57
366 h	0.0	10.02	1.88 × 10^−4^	35.14	4.68 × 10^−4^	4.23 × 10^4^	–
	2.0	32.93	7.59 × 10^−5^	860.6	1.94 × 10^−5^	1.85 × 10^5^	77.13
	5.0	41.94	5.66 × 10^−5^	166.5	6.46 × 10^−6^	3.31 × 10^5^	87.22
